# Development and effectiveness of a mobile-based autonomy support program for the prevention of metabolic syndrome in middle-aged women

**DOI:** 10.3389/fpubh.2024.1334988

**Published:** 2024-02-07

**Authors:** Miseon Seo, Eun-Young Jun, Hyunjin Oh

**Affiliations:** ^1^Korea Institute of Oriental Medicine (KIOM), Daejeon, Republic of Korea; ^2^Department of Nursing, Daejeon University, Daejeon, Republic of Korea; ^3^College of Nursing, Gachon University, Incheon, Republic of Korea

**Keywords:** health behavior, metabolic syndrome, mobile application, middle aged, personal autonomy

## Abstract

**Objective:**

Utilizing self-directed strategies for maintaining and managing healthy lifestyle habits is efficient, and it is essential to consider individual motivation, as it is a factor that directly influences the adoption and maintenance of healthy behaviors. The study aimed to assess the effects of a mobile-based autonomy support program on basic psychological needs, autonomous motivation, health behavior, and metabolic syndrome indicators in middle-aged women.

**Methods:**

This study was a non-randomized controlled trial with a pre-test and post-test design, focused on validating a mobile-based autonomy-supportive program to prevent metabolic syndrome in middle-aged women. The experimental group participated in a 12-week mobile-based autonomy support program, which included components such as education, physical activity guidance, dietary management, and real-time data monitoring. In contrast, the control group was provided with comparable educational resources. Assessments of basic psychological needs, autonomous motivation, health behavior, and metabolic syndrome indicators were conducted at baseline and again at the 12-week mark.

**Results:**

After a 12-week period, the experimental group demonstrated significant enhancements in autonomy (*p* = 0.004) and competence (*p* < 0.001), two key dimensions of basic psychological needs. Autonomous motivation (*p <* 0.001) and health behavior scores (*p <* 0.001) were also significantly higher in the experimental group, while waist circumference (*p* = 0.048) and systolic blood pressure (*p* = 0.011) were significantly reduced. Other variables such as relatedness, high-density cholesterol, fasting blood sugar, diastolic blood pressure, and neutral fat scores were also improved in the experimental group, but these changes were not statistically significant.

**Conclusion:**

The autonomy support program offers a cost-effective and community-accessible health care strategy for middle-aged women and may be integrated into various nursing practices.

## Introduction

Metabolic syndrome is a metabolic disorder that increases the risk of cardiovascular disease, characterized by the presence of at least three of the five risk factors: abdominal obesity, hypertension, hypertriglyceridemia, low high-density lipoprotein, and insulin resistance (1, 2). The prevalence of metabolic syndrome in Korea is 17.6% in the 40s, but increases with age, being 22.4% in the 50s, 35.5% in the 60s, and 43.3% in the 70s (3). Considering the impact of modern lifestyle factors, such as high-fat diets and lack of exercise (1), along with the rising prevalence of hypertension and diabetes, the overall risk for metabolic syndrome in the population is expected to rise. Middle-aged women often experience reduced opportunities for physical activity due to responsibilities such as work, household chores, and childcare. Additionally, hormonal fluctuations associated with menopause can contribute to both physical and mental health challenges, which have been documented to influence the risk of metabolic syndrome (4). The frequency of metabolic syndrome is significantly higher with age, increasing the risk of cardiovascular diseases (5). The growing risk of chronic conditions, like abdominal obesity (1), emphasizes the need for programs to prevent metabolic syndrome from pre-menopause (4).

The prevention of metabolic syndrome should focus not merely on reducing weight or waist circumference but on improving lifestyle habits centered on diet and exercise, with long-term continuous practice of healthy behaviors being paramount. Healthy behaviors refer to holistic activities related to the maintenance and promotion of health (6) and typical healthy behaviors for metabolic syndrome include not only increased physical activity and healthy eating habits but also weight control, moderation in drinking, smoking cessation, stress management, regular health check-ups, and adequate sleep and rest (1).

Modifying lifestyle habits is challenging as it requires individual willpower and long-term consistent management to see effects (7). Therefore, to maintain and manage healthy lifestyle habits, utilizing self-directed strategies is efficient (8) and consideration must be given to individual motivation, a factor that directly influences healthy behaviors (9).

Deci and Ryan (10) have explained that motivation is effective in sustaining individual behavioral changes for carrying out healthy behaviors, and through Self-Determination Theory (SDT), they illustrate how the satisfaction of three basic psychological needs—autonomy, competence, and relatedness—promotes autonomous motivation formed internally, influencing the initiation and persistence of actions. Moreover, the SDT-based model of health behavior change posits that an autonomy-supportive health care environment, individual inclination toward autonomy, and internal and external life goals affect the satisfaction of the three basic psychological needs, contributing to the maintenance of physical and mental health (11). Among these, while individual inclination toward autonomy and internal and external life goals may be difficult to change through intervention, the autonomy-supportive health care environment is a changeable factor through various intervention methods by health professionals, including nurses (12, 13). Particularly, the autonomy-supportive health care environment, as a changeable environmental condition, enhances autonomous motivation formed internally by influencing the satisfaction of basic psychological needs, thereby sustaining healthy behaviors and being a crucial element for mental and physical health (10, 11).

Research on autonomy-supportive health care environments by health professionals has been described across various areas, including reduction in medication, improved fitness, physical form, increased general well-being for diabetes patients (14), weight loss and maintenance in exercise programs for the obese (12, 13), and changes in blood pressure, triglycerides, and fasting blood sugar (15). Also, it has been identified as a major variable that significantly influences self-care in osteoarthritis and coronary artery intervention patients, enhancing healthy behaviors (16, 17). Although these interventions have positive effects, their implementation poses challenges for middle-aged women who find it difficult to participate regularly due to household chores and childcare responsibilities, as most programs are face-to-face and conducted at specific locations. Therefore, an intervention that considers the characteristics of middle-aged women and barriers to exercise, and that is easily accessible, is needed. Nursing interventions using mobile technology have advantages of accessibility without the temporal and spatial constraints of traditional face-to-face programs (17), and have been shown to be effective in promoting physical activity (18, 19). Smooth communication between the target individuals and healthcare providers also enhances monitoring and self-management, improving symptoms of chronic diseases (20).

**Figure 1:**
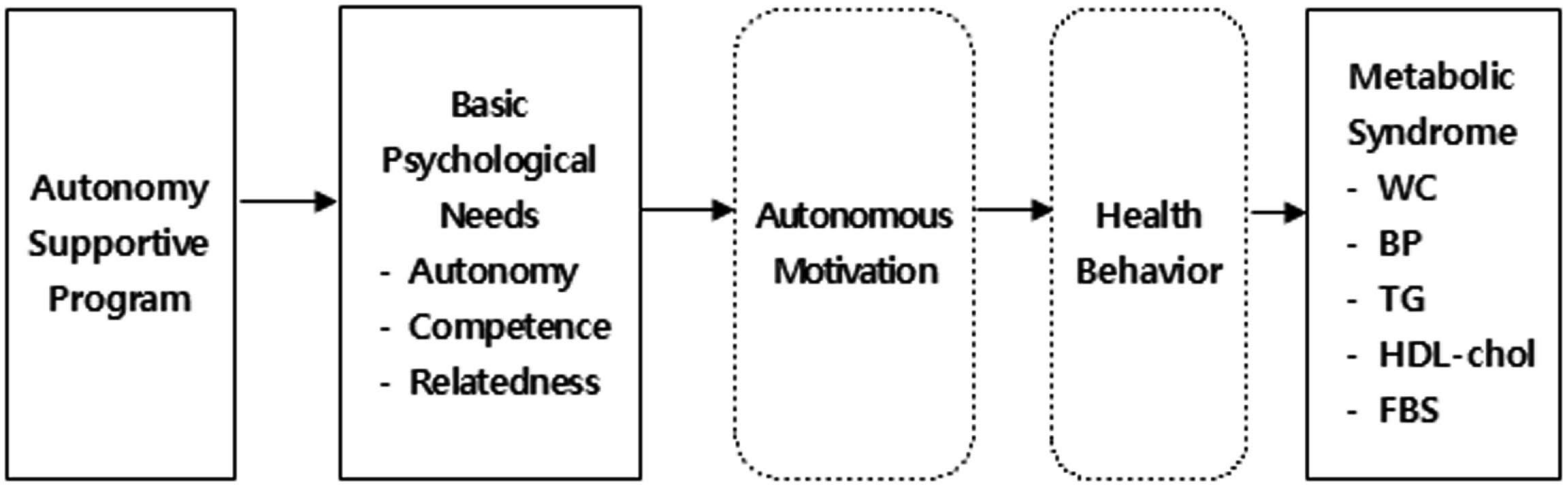
Research framework. WC, Waist Circumference; BP, Blood Pressure; TG, Triglyceride; HDL-Chol, High Density Lipoprotein Cholesterol; FBS, Fasting Blood Sugar.

The purpose of this study is to develop and implement a mobile-based autonomy-supportive program for the prevention of metabolic syndrome in middle-aged women, and to subsequently validate the effects of this mobile-enabled autonomy-supportive program on their basic psychological needs, autonomous motivation, health behaviors, and physiological indicators of metabolic syndrome. The conceptual framework outlining the study design and the primary variables is depicted in Figure 1.

## Materials and methods

### Study design, participants, and setting

The study received ethical approval from Daejeon University (1040647-201806-HR-027-02). In consideration of ethical principles, the same educational materials and gift certificates were provided to the control group after the intervention with the experimental group was completed.

Participants were recruited from a tertiary hospital’s internal medicine outpatient clinic (D Hospital) and a public health center (H public health center) located in (Chungcheong-do, South Korea). Inclusion criteria for the study were having 1–2 of the five risk factors for metabolic syndrome, such as waist circumference over 85 cm, systolic blood pressure over 130 mmHg, diastolic pressure over 85 mmHg, triglycerides over 150 mg/dL, high-density lipoprotein cholesterol under 50 mg/dL, or fasting blood sugar over 100 mg/dL (2, 3). Exclusion criteria included individuals with musculoskeletal diseases like arthritis that would impede continuous exercise, those with serious diseases like cancer, heart or kidney disease, and individuals taking medication or antidepressants that might affect weight and blood pressure.

**Figure 2:**
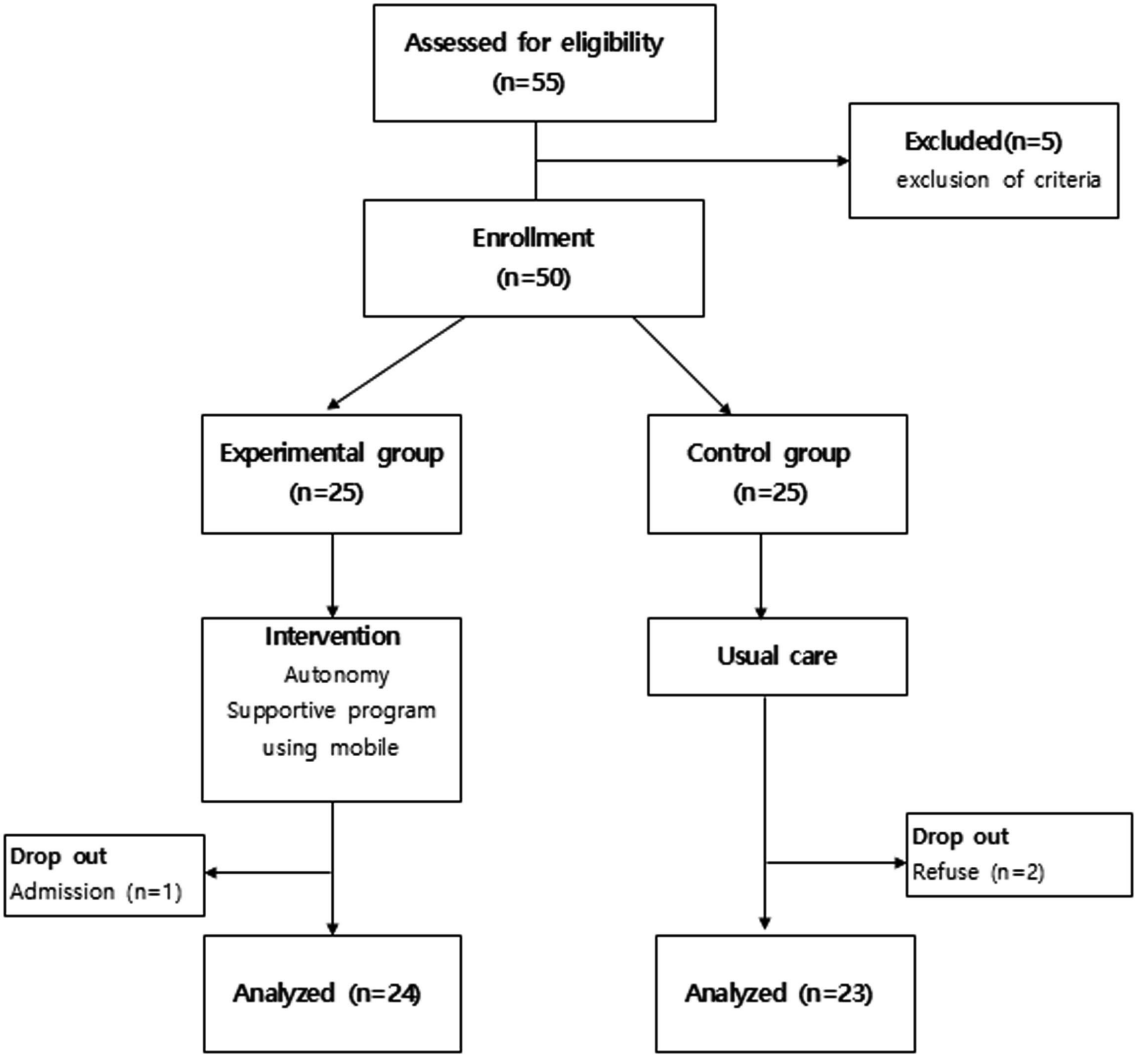
Number of patients included.

The required sample size of 50 was calculated using the G power 3.1.9 program, based on research by Silva et al. (21), with a significance level of *α* = 0.05, test power of 0.80, and effect size of 0.95. Anticipating a 30% attrition rate, an initial sample size of 25 participants was chosen for each of the experimental and control groups. The experimental group consisted of individuals recruited from the outpatient Internal Medicine department at D Hospital, while the control group was composed of participants sourced from H public health center, a setting with comparable environmental and socioeconomic conditions. During the study, one participant from the experimental group withdrew in the fourth week due to hospitalization from a traffic accident, and two participants from the control group withdrew due to personal reasons and were unable to participate in the post-intervention survey. Therefore, the final analysis was conducted with data from 24 participants in the experimental group and 23 participants in the control group (Figure 2).

### Measures

#### Basic psychological needs

The basic psychological needs were measured using the Basic Psychological Needs Scale developed by La Guardia et al. (22), and translated by Lee and Park (23). The instrument includes 21 items across three subscales: autonomy (seven items), competence (six items), and relatedness (eight items), each rated on a seven-point Likert scale from “not at all true” (1 point) to “very true” (7 points). The maximum scores are 49 for autonomy, 42 for competence, and 56 for relatedness.

#### Autonomous motivation

For the measurement of behavioral motivation types, the study utilized eight items related to autonomous motivation from the Treatment Self-Regulation Questionnaire developed by Ryan and Connell (24), translated by Park (16). Permission was secured for tool use. It consists of eight items for measuring autonomous motivation, each scored on a seven-point Likert scale ranging from “not at all true” (1) to “very true” (7), where higher scores reflect greater autonomous motivation, with a total possible score of 56.

#### Health behaviors

The instrument to assess health behavior in this study was developed by Kang (25), specifically to evaluate the lifestyle habits of individuals with metabolic syndrome. The tool assesses lifestyle behaviors across six areas: physical activity/weight control, dietary habits, alcohol/tobacco use, sleep/relaxation, stress management, and health check-ups. Using a four-point scale (“never” = 1 to “always” = 4), higher scores denote healthier behaviors. It includes 36 questions, each with a maximum of four points, totaling a maximum score of 144.

#### Physiological indicators of metabolic syndrome

- Waist circumference is measured with the feet spread approximately 25–30 cm apart to evenly distribute weight, and while breathing out comfortably, keeping the measuring tape horizontal to the floor. The measurement is taken at the midpoint between the lowest point of the ribs and the highest point of the pelvis (iliac crest).
- Blood pressure is measured in the right upper arm using an automatic blood pressure monitor (Omron) after allowing at least 10 min of sufficient rest. The measurement is taken twice, with a 5-min interval between readings, and the average value is used.
- Blood tests for triglycerides (TG), high-density lipoprotein cholesterol (HDL), and fasting blood glucose (FBS) were conducted by drawing 4 mL of blood from the brachial vein between 9 and 10 AM on the day of the test, following the participants’ maintenance of a fasting state for over 9 h as instructed by the researchers the previous day (1, 7). The collected specimens were placed into plain tubes and centrifuged at 3,500 rpm for 10 min to separate the plasma. Blood analysis was carried out by clinical pathologists in the diagnostic laboratory departments.

### Procedures

#### Pretest

After obtaining written consent, the research assistant distributed questionnaires on general characteristics, basic psychological needs, autonomous motivation, and health behavior for self-completion, and physiological indicators of metabolic syndrome were collected.

#### Intervention

In this study, the principal researcher (PI) conducted the mobile-based autonomy support program, and data collection was handled by a research assistant (RA) under the researcher’s supervision. To ensure the internal validity of the experimental intervention, the education was led by PI, and the collection of survey data was done by one RA.

**Figure 3:**
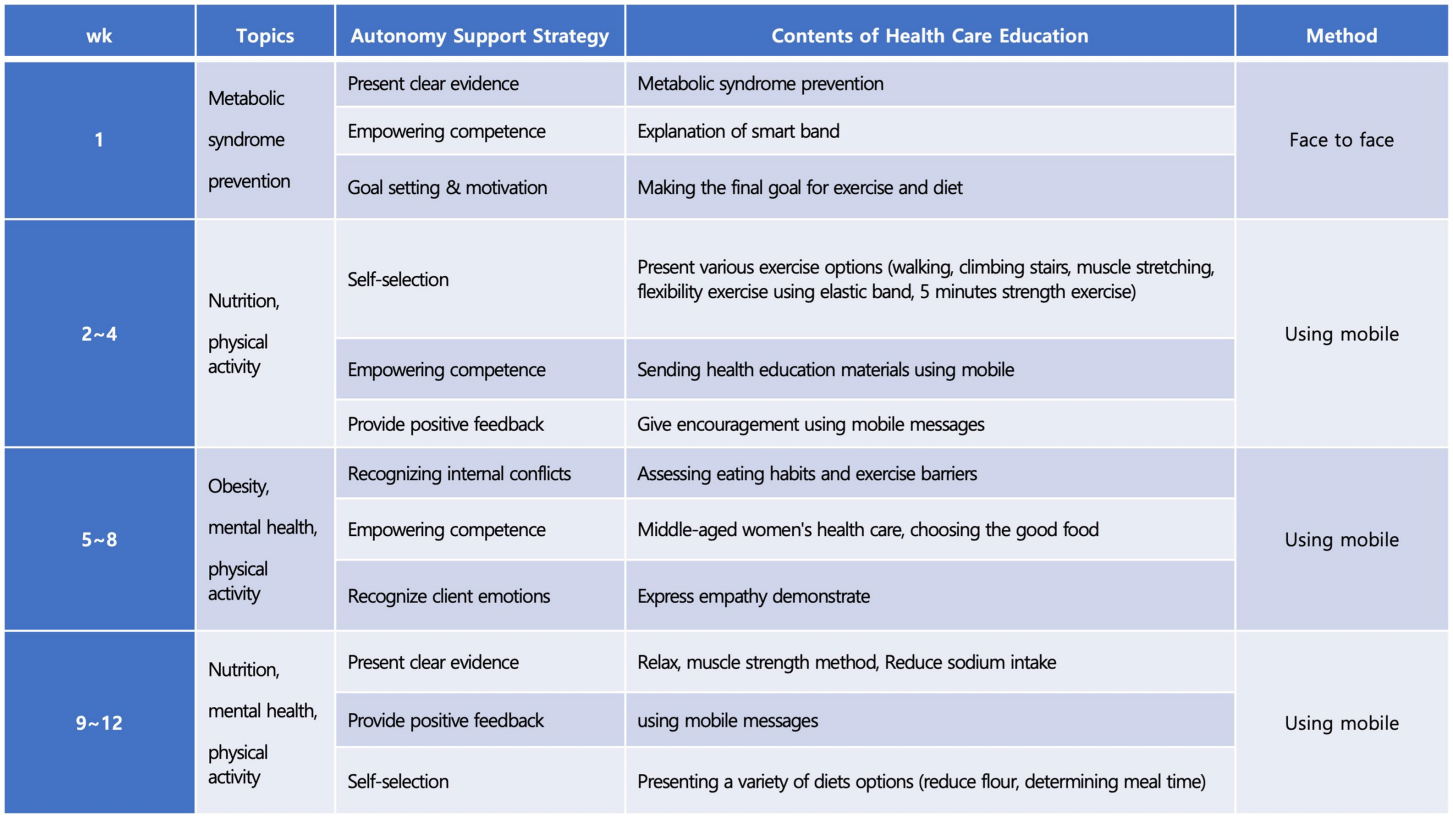
Intervention components.

The experimental group, after a preliminary survey, underwent the mobile-based autonomy support program for 12 weeks (Figure 3). They were provided with educational materials as PPTs developed for the prevention and management of metabolic syndrome, including an understanding of the disease, its causes, diet management, exercise therapy, and lifestyle therapy. Goals were set with participants regarding types and times of exercise, daily total steps, meal times, and motivation support was provided. Stretching instruction posters and beginner’s resistance bands were given for exercise practice, and a wearable smart band was worn continuously on the wrist. An application for the smart band was installed on mobile phones, linked via Bluetooth. Real-time data collected through the smart band, including step count, heart rate, sleep patterns, and exercise records, were transmitted to the researcher through mobile SNS according to a protocol. Feedback and monitoring were provided by PI through mobile SNS, following different phases of the program (1–4 weeks, 5–8 weeks, and 9–12 weeks). Targets for daily total steps, physical activity, and meal times were set and maintained with motivation. The PI also provided health education and feedback via mobile SNS once a week and conducted phone counseling when needed.

The control group was given educational materials as PPTs for metabolic syndrome prevention and management and once provided explanations via face to face about the disease, its causes, diet management, exercise therapy, and lifestyle therapy, mirroring the approach with the experimental group after the pretest.

#### Posttest

The RA, who facilitated the pretest, distributed the questionnaires for self-administration to the participants and collected them upon completion. Upon conclusion of the intervention and after the post-intervention survey, the control group received the same health education materials that were used in the experimental group, delivered via mobile platforms, in adherence to ethical considerations.

### Statistical analyses

The collected data were analyzed using the SPSS Win 22.0 Program as follows: general characteristics of the participants were examined using frequency analysis, and were analyzed using means, standard deviations, real numbers, and percentages. Homogeneity tests according to general characteristics were conducted using the χ^2^-test and *t*-test, while pre-homogeneity testing for dependent variables was done with the *t*-test. Normality testing for dependent variables was confirmed with the Kolmogorov–Smirnov test, and variables satisfying normal distribution were analyzed using the *t*-test. The analysis of *post hoc* mean differences for dependent variables was conducted using the *t*-test. Additionally, the changes before and after the experiment in both the experimental and control groups were assessed with the paired *t*-test.

## Results

### Baseline characteristics

The study included 47 participants, with 24 in the experimental group and 23 in the control group. The homogeneity analysis of general and health-related characteristics revealed no significant differences between the two groups. The same applied to dependent variables such as basic psychological needs, autonomous motivation, health behaviors, and physiological indicators of metabolic syndrome. This confirmed that both groups were homogeneous (Table 1).

**Table 1:** Homogeneity of characteristics and dependent variables (*N* = 47).

Variables	Categories	Exp. (*n* = 24)	Cont. (*n* = 23)	*t*	*p*
		*n* (%) or M ± SD			
Age (year)	40 ∼ 45	5 (20.8)	4 (17.4)	−0.73	0.469
	46 ∼ 50	13 (54.2)	13 (56.5)		
	51 ∼ 60	6 (25.0)	6 (26.1)		
		48.08 ± 3.28	49.0 ± 5.16		
Education level	≤Middle school	5 (20.8)	5 (20.8)	4.23	0.213
	High school	13 (54.2)	13 (54.2)		
	≥University	6 (25.0)	6 (25.0)		
Marital status	Married	22 (91.7)	18 (78.3)	2.04	0.563
	Other	2 (8.4)	5 (21.7)		
Occupation	Yes	10 (41.7)	15 (65.8)	9.05	0.060
	No	14 (58.3)	8 (34.8)		
Drinking	Yes	19 (79.2)	12 (56.5)	5.09	0.165
	No	5 (20.8)	11 (47.8)		
Regular exercise	Yes	7 (29.2)	4 (17.4)	1.73	0.188
	No	17 (70.8)	19 (82.6)		
Basic psychological needs					
Autonomy		35.21 ± 3.04	36.30 ± 2.18	−1.42	0.164
Competence		30.42 ± 1.98	31.43 ± 1.75	−1.87	0.069
Relatedness		40.46 ± 3.36	41.52 ± 2.15	−1.21	0.233
Autonomous motivation		43.08 ± 2.72	41.87 ± 2.63	1.55	0.127
Health behavior		2.81 ± 0.20	2.80 ± 0.14	0.33	0.742
Physiological indicators of metabolic syndrome					
WC (㎝)		84.57 ± 2.36	84.86 ± 2.33	−0.42	0.678
SBP (㎜Hg)		134.83 ± 12.16	131.96 ± 13.22	0.78	0.441
DBP (㎜Hg)		80.42 ± 7.22	79.04 ± 7.77	0.63	0.533
TG (㎎/㎗)		147.33 ± 38.36	167.22 ± 43.13	−1.67	0.102
HDL-Chol (㎎/㎗)		43.88 ± 5.13	46.83 ± 6.13	−1.79	0.080
FBS (㎎/㎗)		96.46 ± 9.22	95.96 ± 8.32	0.20	0.846

Exp., Experimental group; Cont., Control group; WC, Waist circumference; SBP, Systolic blood pressure; DBP, Diastolic blood pressure; TG, Triglyceride; HDL-Chol, High density lipoprotein cholesterol; FBS, Fasting blood sugar.

### Effects of autonomy support program on dependent variables

In the experimental group, significant improvements were observed in autonomy (36.17 ± 2.66 vs. 33.87 ± 2.56, *t* = 3.01, *p* = 0.004) and competence (31.58 ± 2.83 vs. 27.57 ± 2.41, *t* = 5.23, *p* < 0.001) among basic psychological needs scores compared to the control group. However, in the control group, autonomy, competence, and relatedness scores were lower differently the experimental group after the 12 weeks.

Autonomous motivation (43.17 ± 2.20 vs. 34.74 ± 2.47, *t* = 12.36, *p* < 0.001) and health behavior scores (124.50 ± 4.21 vs. 111.61 ± 5.15, *t* = 9.41, *p* < 0.001) were also significantly higher in the experimental group. On the other hand, in the control group autonomous motivation score was decreased, and health behavior score lower increase than the experimental group.

Physiological indicators like waist circumference (83.48 ± 2.35 vs. 84.81 ± 2.13, *t* = ¬2.04, *p* = 0.048) and systolic blood pressure (129.29 ± 7.92 vs. 135.13 ± 7.16, *t* = ¬2.65, *p* = 0.011) were significantly reduced in the experimental group. However, diastolic blood pressure, neutral fat and fasting blood sugar scores were lower in the experimental group but not statistically significant, and high-density cholesterol score was higher, these were not statistically significant. In the control group, systolic blood pressure, diastolic blood pressure, and fasting blood sugar scores were increased after 12 weeks (Table 2).

**Table 2:** Effects of autonomy support program on dependent variables (*N* = 47).

Variables	Group	Pre-test	Post-test	Paired test		Difference	*t*	*p*
		M ± SD		*t*	*p*	(post-pre)		
Basic psychological needs								
Autonomy	Exp. (*n* = 24)	35.21 ± 3.04	36.17 ± 2.66	0.94	0.354	0.96 ± 4.96	3.01	0.004
	Cont. (*n* = 23)	36.30 ± 2.18	33.87 ± 2.56	4.26	<0.001	−3.09 ± 3.48		
Competence	Exp. (*n* = 24)	30.42 ± 1.98	31.58 ± 2.83	1.58	0.127	1.17 ± 3.61	5.23	<0.001
	Cont. (*n* = 23)	31.43 ± 1.75	27.57 ± 2.41	6.37	<0.001	−3.87 ± 2.91		
Relatedness	Exp. (*n* = 24)	40.46 ± 3.36	41.63 ± 2.67	1.19	0.245	1.17 ± 4.79	1.73	0.091
	Cont. (*n* = 23)	41.52 ± 2.15	40.22 ± 2.92	1.78	0.088	−0.16 ± 0.44		
Autonomous motivation	Exp. (*n* = 24)	43.08 ± 2.72	43.17 ± 2.20	0.12	0.905	0.08 ± 3.39	12.36	<0.001
	Cont. (*n* = 23)	41.87 ± 2.63	34.74 ± 2.47	9.04	<0.001	−7.13 ± 3.78		
Health behavior	Exp. (*n* = 24)	101.33 ± 7.11	124.50 ± 4.21	14.26	<0.001	23.17 ± 9.03	9.41	<0.001
	Cont. (*n* = 23)	100.74 ± 4.92	111.61 ± 5.15	6.65	<0.001	10.87 ± 7.84		
Physiological indicators of metabolic syndrome								
WC (㎝)	Exp. (*n* = 24)	84.57 ± 2.36	83.48 ± 2.35	10.03	<0.001	−1.09 ± 0.53	2.04	0.048
	Cont. (*n* = 23)	84.86 ± 2.33	84.81 ± 2.13	0.26	0.795	−0.04 ± 0.79		
SBP (㎜Hg)	Exp. (*n* = 24)	134.83 ± 12.16	129.29 ± 7.92	4.23	<0.001	−5.54 ± 6.41	2.65	0.011
	Cont. (*n* = 23)	131.96 ± 13.22	135.13 ± 7.16	1.67	0.109	3.17 ± 9.12		
DBP (㎜Hg)	Exp. (*n* = 24)	80.42 ± 7.22	78.96 ± 4.63	0.92	0.369	−1.46 ± 7.80	1.36	0.182
	Cont. (*n* = 23)	79.04 ± 7.77	81.09 ± 6.07	2.04	0.241	2.04 ± 8.13		
TG (㎎/㎗)	Exp. (*n* = 24)	147.33 ± 38.36	145.52 ± 35.4	0.87	0.394	−2.21 ± 12.45	1.04	0.306
	Cont. (*n* = 23)	167.22 ± 43.13	160.52 ± 38.1	2.69	0.013	−6.70 ± 11.92		
HDL-Chol (㎎/㎗)	Exp. (*n* = 24)	43.88 ± 5.13	46.75 ± 3.87	4.47	<0.001	2.88 ± 3.15	1.15	0.258
	Cont. (*n* = 23)	46.83 ± 6.13	45.39 ± 4.25	1.59	0.125	−1.43 ± 4.32		
FBS (㎎/㎗)	Exp. (*n* = 24)	96.46 ± 9.22	94.17 ± 9.55	2.07	0.050	−2.29 ± 5.42	0.63	0.529
	Cont. (*n* = 23)	95.96 ± 8.32	96.09 ± 11.17	0.10	0.919	0.13 ± 6.12		

Exp., Experimental group; Cont., Control group; WC, Waist circumference; SBP, Systolic blood pressure; DBP, Diastolic blood pressure; TG, Triglyceride; HDL-Chol, High density lipoprotein cholesterol; FBS, Fasting blood sugar.

## Discussion

This 12-week study implementing a mobile-based autonomy support program showed statistically significant differences in the basic psychological needs of autonomy and competence, but not relatedness. These results align with previous research, suggesting that healthcare environments supporting autonomy and competence are effective (11, 26). Similar findings have been reported in other programs for patients with diabetes (14) and osteoarthritis patients (17). Autonomy, as the belief in oneself as the agent and regulator of behavior (10), can be effectively enhanced through goal setting and allowing self-choice, without the feeling of being controlled (15). In this study, the participants were guided to set health goals and choose suitable exercise methods autonomously, without feeling controlled. Strategies such as using mobile devices for information provision to enhance competence and using smart bands for self-monitoring proved effective, supporting related studies (18). Yet, a study by Park (16) revealed that coronary intervention patients needed autonomy-supported feedback to feel interest in health behavior, lower than in autonomy and relatedness. Thus, the provision of continuous feedback based on physical activity via mobile SNS in this study seems to be an appropriate strategy.

In this study, no significant change was noted in relatedness, consistent with previous research on autonomy support programs (15, 17). Relatedness, the desire to create meaningful connections with others (10), was targeted by using mobile technology for positive feedback and encouragement. A previous study (17) showed enhancement in relatedness through the mobile app. This suggests that using a communal chat room might be more effective than one-on-one methods for improving relatedness, indicating a need for various intervention strategies in future research.

Autonomous motivation is a vital factor influencing the initiation and persistence of health behavior (11). In our study, the experimental group maintained autonomous motivation scores, while the control group decreased, showing a significant difference between the two. Research by Jay et al. (27) showed a significant effect in the experimental group’s autonomous motivation after a year-long weight management intervention based on self-determination theory. This aligns with Schmidt et al. (14), where lifestyle changes-related autonomous motivation meaningful increased through autonomy-supportive strategies. Compared to a one-year focus on voluntary exercise control and intrinsic enjoyment, this study’s 12-week intervention may be too short to observe internalized changes in autonomous motivation toward health behavior. Long-term intervention research is deemed necessary for analyzing the effects over time.

The health behaviors in this study can be considered to have persisted through autonomous motivation, based on self-determination theory and satisfaction of basic psychological needs. Both the experimental and control groups showed an increase post-intervention, and there was a significant difference in the degree of change in health behavior scores between the two groups. This program focused on recognizing the need for health behavior through autonomy-supportive strategies, exploring and autonomously selecting appropriate physical activity enhancement methods, thus encouraging individuals to sustain health behavior on their own. These findings are consistent with previous research showing that interventions supporting autonomy are effective in improving dietary control in diabetes patients (26) and promoting physical activity in adult women (18).

Modifying lifestyle habits for the prevention of metabolic syndrome is challenging as it requires consistent management with individual will (4). In this study, a mobile SNS was used as an information-providing strategy to support the clear foundation of an autonomy-supportive program, offering health education and feedback on nutrition, physical activity, obesity, and mental health. This can be seen as corroborating research that found higher motivation for metabolic syndrome health behavior when more information about health behavior was available (9). In a metabolic syndrome-targeted lifestyle intervention study (28), a group prescribed with habits tailored to health needs every 2 weeks showed a significant increase in the practice rate of health behavior compared to a control group given only basic information. These findings suggest that future research should explore and develop various educational intervention methods to provide appropriate information tailored to health needs instead of uniformly providing the same information to all subjects.

This study revealed statistically significant differences in waist circumference and systolic blood pressure among physiological indicators of metabolic syndrome. These research results can be explained by various outcomes from multiple studies. For example, a web-based autonomy-supportive program targeting postmenopausal women showed significant effects on waist circumference, neutral fat, and systolic blood pressure (29), while a motivation-enhanced weight loss program showed statistically significant differences in blood pressure and neutral fat 3 months later (15). A daily life-based physical activity enhancement program (18) showed a significant decrease in fasting blood sugar after a 12-week intervention but no significant changes in total cholesterol and high-density cholesterol levels. Such physiological indicators of metabolic syndrome have shown different results in various studies. According to meta-analysis, physiological indicators such as weight, blood sugar, waist circumference decrease, and high-density cholesterol increase are reported when doing aerobic exercise for more than 60 min, using many muscles, on average for 16 weeks (30). Therefore, based on the research results (29) that suggest the need for regular exercise and lifestyle changes for more than 12 weeks in education programs targeting cardiometabolic risk in postmenopausal women, there is a need to repeatedly measure changes in physiological indicators through long-term observation. Although this study may differ from standardized exercise intensity or type in existing exercise programs by focusing on supporting the autonomy of the subjects to choose the intensity or type of exercise through autonomous motivation, it is judged to have a positive impact on the prevention of metabolic syndrome in middle-aged women through changes in waist circumference and systolic blood pressure. To verify changes in the physiological indicators of metabolic syndrome, considering the relatively short intervention period of 12 weeks in this study, it is necessary to apply the program by extending the intervention period in the future, and post-intervention repeated measurements are required to verify the sustained effects after the program’s completion.

In summary, a 12-week mobile program based on self-determination theory significantly improved autonomy, competence, motivation, health behaviors, waist circumference, and systolic blood pressure in middle-aged women at risk for metabolic syndrome. However, no significant changes were observed in relatedness, diastolic blood pressure, or other lipid profiles. Recommendations include extending the intervention period for more robust results and conducting follow-up studies for long-term effects, ideally in collaboration with community health centers or hospitals.

## Conclusion and recommendations

This study demonstrated that a 12-week mobile intervention program, grounded in self-determination theory, effectively enhanced autonomy, competence, motivation, and health behaviors, and led to significant reductions in waist circumference and systolic blood pressure among middle-aged women at risk of metabolic syndrome. No notable changes were observed in relatedness, diastolic blood pressure, or lipid profiles. For more definitive outcomes, it is recommended to extend the duration of the intervention and conduct longitudinal studies to assess the long-term effects. Such studies would benefit from partnerships with community health centers or hospitals for broader implementation and support.

## Data availability statement

The original contributions presented in the study are included in the article/supplementary material, further inquiries can be directed to the corresponding authors.

## Ethics statement

The studies involving humans were approved by Daejeon University Institutional review board. The studies were conducted in accordance with the local legislation and institutional requirements. The participants provided their written informed consent to participate in this study.

## Author contributions

MS: Conceptualization, Data curation, Formal Analysis, Investigation, Methodology, Resources, Writing – original draft. E-YJ: Conceptualization, Formal Analysis, Methodology, Supervision, Writing – original draft, Writing – review & editing. HO: Supervision, Writing – original draft, Writing – review & editing.
